# *MT-ATP6* 9035T>C Variant Causes Ataxia With Azoospermia and Apparent Anticipation in a Four-generation Kindred

**DOI:** 10.1007/s12311-026-02008-z

**Published:** 2026-04-25

**Authors:** Changrui Xiao, David Zhu, Jon Pryor, Sally A. Frutiger, H. Brent Clark, Hannah L. Casey, Matthew Bower, Guo-yun Yu, Xiaofei Du, Camilo Toro, Christopher M. Gomez

**Affiliations:** 1 Department of Neurology, University of California Irvine, Orange, CA 92868 USA; 2 Department of Neurology, University of Chicago, Chicago, IL 60637 USA; 3 Department of Urology, University of Minnesota, Minneapolis, MN 55455 USA; 4 Department of Neurology, University of Minnesota, Minneapolis, MN 55455 USA; 5 Department of Lab Medicine and Pathology, University of Minnesota, Minneapolis, MN 55455 USA; 6 National Human Genome Research Institute (NHGRI), Bethesda, MD 20892-2152 USA

**Keywords:** Mitochondria, Heteroplasmy, Ataxia, Azoospermia, MTATP6

## Abstract

**Background:**

The hereditary cerebellar ataxias are a clinically and genetically heterogeneous group of disorders characterized by progressive cerebellar degeneration leading to incoordination of gait, speech, limb, and eye movements. Hundreds of genes encoding diverse proteins underlie this family of degenerative disorders that may exhibit autosomal dominant, recessive, mitochondrial, or X-linked inheritance. Variations in clinical presentation, such as age of onset and severity, are typical of these disorders and are attributed to other genetic effects, notably repeat expansion instability.

**Methods:**

In this observational study, we evaluated 45 individuals in a 5-generation kindred exhibiting features of progressive ataxia and cognitive impairment with wide-ranging ages of onset, intergenerational anticipation, diverse clinical features, and male infertility.

**Results:**

The variant (m.9035 T > C) in the gene *MT-ATP6* was detected in all affected individuals, and the age of onset from early childhood to the 8th decade was inversely correlated with heteroplasmy levels. Neuropsychological evaluation of affected individuals demonstrated low average/borderline overall intellectual ability and weaknesses in working memory, executive function, memory, and fine motor skills.

Affected males had testicular atrophy and azoospermia. Postmortem examination revealed widespread cerebellar Purkinje cell loss. Testicular biopsy from one sterile male demonstrated a complete absence of germ cells and progenitors.

**Discussion:**

This study expands the phenotypic spectrum of *MT-ATP6* to include azoospermia in affected males with cerebellar ataxia. Those with low levels of heteroplasmy had mild adult-onset ataxia reminiscent of many forms of SCA. Those with high levels of heteroplasmy had early onset and suffered from the full complement of ataxia, mild cognitive impairment, and in males’ azoospermia.

**Supplementary Information:**

The online version contains supplementary material available at 10.1007/s12311-026-02008-z.

## Introduction

The hereditary cerebellar ataxias (SCAs) are a clinically and genetically heterogeneous group of neurological disorders characterized by progressive gait ataxia, limb incoordination, and dysarthria due to degeneration of the cerebellum and its connections [1, 2]. To date, the ataxias have been attributed to mutations in hundreds of autosomal or X-linked genes. These mutations give rise to dominantly or recessively inherited degenerative ataxia syndromes, ranging widely in age of onset, extracerebellar manifestations, and severity. Phenotypic variations within individual ataxia genotypes or families are attributed to allelic variation, repeat expansion instability, or modifier genes. In addition to nuclear-encoded genes causing ataxia, mutations in mitochondrial-encoded genes are also responsible for a wide array of neurological syndromes, including cerebellar ataxias. Phenotypic variability in diseases due to mitochondrial DNA mutations may additionally be related to variations in heteroplasmy manifestation [3].

*MT-ATP6* is a mitochondrial gene that encodes a subunit of complex V bound to the inner membrane [4]. *MT-ATP6* was one of the first mitochondrial genes to be associated with disease over thirty years ago, originally linked to Leigh syndrome and NARP (neuropathy, ataxia, and retinitis pigmentosa) syndrome due to a recurrent pathogenic variant, m.8993 T > G [5]. Currently, 19 different variants in *MT-ATP6* have been reported to be associated with a wide range of phenotypes in addition to Leigh syndrome and NARP, including hereditary sensorimotor neuropathy, cerebellar ataxia, periodic paralysis, cardiomyopathy, spastic paraplegia, intellectual disability, epilepsy, and nephropathy [6, 7]. Two variants, m.9185 T > C and m.9035 T > C, have been reported to cause adult onset cerebellar ataxia without additional features similar to the classic recognized forms of autosomal dominant SCA [8].

Here we describe a 5-generation kindred with progressive ataxia associated with cognitive impairment attributed to a mutation (m.9035 T > C) in *MT-ATP6.* We reviewed the literature for phenotype, genotype, imaging, and biochemical data on previously published cases with the same variant. Male infertility may be an underrecognized phenotype of this condition. The disorder has a wide range of age of onset, from early childhood to the 8th decade, and is inherited only from affected or presymptomatic females. The successively earlier ages of onset in offspring are attributable to increasing proportions of heteroplasmy in the *MT-ATP6* variant.

## Methods

### Literature Review

A targeted literature review was conducted to identify publications describing patients harboring the MT-ATP6 m.9035 T > C (p.Leu170Pro) variant. Searches were performed in PubMed using combinations of the following terms: *“MT-ATP6”*, *“ATP6”*, *“m.9035 T* > *C”*, *“9035 T* > *C”*, and *“Leu170Pro.”* The search was supplemented by reviewing variant-specific entries in ClinVar and by manually screening the reference lists of relevant articles, such as reviews, to identify additional reports.

Eligible studies included case reports, case series, or cohort studies that reported at least one individual with the MT-ATP6 m.9035 T > C variant and provided clinical or phenotypic information on an individual level. Review articles, purely functional studies without a described patient, and publications lacking sufficient variant-level detail were excluded unless they contained original patient data.

For each eligible study, data were extracted regarding the number of reported individuals with the variant, clinical phenotype, age at onset, heteroplasmy when available, and other relevant clinical findings. When cohort studies included multiple MT-ATP6 variants, only individuals specifically reported with the m.9035 T > C variant were counted in the analysis. Duplicate patient descriptions across publications were assessed by comparing clinical details, family structure, and institutional origin to minimize double counting.

### Clinical Data

Phenotypic data, including neurological exam and brain imaging, and biochemical data, were collected as part of a research protocol [9] on 2 individuals. Additionally clinical data was on 43 additional individuals based on availability via clinical evaluation when possible, historical medical records, or phone interview. All are part of an expanded kindred with hereditary cerebellar ataxia. To account for the possibility that individuals may be presymptomatic, we recorded the current age for any reportedly unaffected individuals. An autopsy, including brain pathology, was performed on one individual. All data was collected at two academic medical centers and the NIH clinical center.

### Cognitive Profile

A subset of thirteen individuals diagnosed with ataxia (Ataxia Group, mean/standard deviation age = 50.15 (12.03); 8 males, 5 females) and five family unaffected family members (Unaffected Group; mean/standard deviation age = 34.6 (10.60); 4 male, 1 female) were administered a brief neuropsychological test battery.

Neuropsychological and emotional function tests (Table 1) included the Weschler Adult Intelligence Scale-Revised (WAIS-R) [10], Rey Auditory Verbal Learning Test (RAVLT) [11], Benton Visual Retention Test (BVRT) [12], Woodcock-Johnson Test of Achievement-Revised (WJ-R: Letter-Word Identification, Passage Comprehension subtests) [13], Verbal Fluency Test [14], Trail Making Tests A& B [15], Finger Tapping Test [15], Grooved Pegboard Test [16], and Brief Symptom Inventory (BSI) [17]. Following standardized test administration, raw test scores were normatively referenced to samples provided in test manuals or chosen for similarity of age/educational level. [10, 11, 13, 18–23].

**Table 1:** Brief description of neuropsychological tests administered

Neuropsychological Test	Description
Wechsler Adult Intelligence Scale-Revised (WAIS-R)	Intellectual ability test which provides a composite estimate of overall intellectual ability (Full Scale Intellectual Quotient; FSIQ) based on several subtests assessing verbal (Verbal Intelligence Quotient; VIQ) and visuospatial (Performance Intelligence Quotient; PIQ) reasoning skills
Digit Span (WAIS-R)	Verbal intellectual ability subtest that assesses the ability to retain and mentally manipulate auditory information for a brief period immediately following presentation
Trailmaking Tests A & B	Executive function task that requires rapid visuomotor execution of a familiar conceptual sequence (Trailmaking Test A) or a more conceptually demanding rapid alternating pattern between two familiar conceptual sequences (Trailmaking Test B)
Rey Auditory Verbal Learning Test (AVLT)	Verbal memory task that assesses learning of a word list over five trials, free recall of list items immediately following presentation, and both recall and recognition of list items following a delay interval
Benton Visual Retention Test (BVRT)	Visual memory task that requires recall of visual geometric designs immediately following presentation
Woodcock-John Tests of Achievement-Revised (WJ-R)	
Letter-Word Identification (WJ-R)	Reading skills task that assesses word recognition skills
Passage Comprehension (WJ-R)	Reading skills task that assesses reading comprehension skills
Verbal Fluency Test (FAS)	Expressive language task that assesses the ability to rapidly produce words based on phonemic (first letter) similarity
Finger Tapping Test	Fine motor skills task that requires repetitive index finger motion to depress a key
Grooved Pegboard Test	Manual dexterity task that requires rapid rotation and placement of pegs into small holes

Although salient, gender-based normative data is generally limited in availability and was not used in this study.

Standardized scores for each test were initially checked to identify any significant deviation from assumption of distribution normality (Shapiro–Wilk Test), independently within each group. Given potential violation of normality assumptions for selected cognitive and motor function tests, the disparity in sample size by group, and the large number of test scores to be evaluated, test scores were: (1) averaged across dominant/non-dominant hands, after initial analysis to rule out handedness effects, (2) evaluated with both parametric (Student T test) and non-parametric (Wilcoxon Signed Rank Test) single sample statistical tests to identify any difference in test performance profile across methods, and (3) identified as statistically significant if meeting criteria established by family wise error rate (Bonferroni correction). Given similar findings across neuropsychological tests when using parametric or nonparametric tests, summary findings were based on parametric test outcomes, independently evaluated within Ataxia and Unaffected Groups.

### Fertility Evaluation

Because of questions of infertility, routine genital exams were performed on six affected males. Serum levels of gonadotrophic and sex hormones were measured in two affected males. Complete semen analysis was performed on seven males. Semen analysis was not performed on one male who had had a prior vasectomy. A bilateral testicular biopsy was performed on one affected male for evaluation of infertility. Formalin-fixed paraffin-embedded tissues were sectioned and subjected to routine hematoxylin and eosin stain.

### Genotyping

Initially, 2 individuals in this cohort underwent research genome/mtDNA sequencing [9] as well as clinical ataxia repeat expansion testing in blood. After the mtDNA m.9035 T > C variant was identified as a candidate variant, blood samples were sent for clinical NGS based testing in a CLIA-certified commercial lab (GeneDx, MD 2021) for confirmation and heteroplasmy level estimation. After confirmation, genotyping for segregation and heteroplasmy analysis was done using DNA extracted from blood from the remaining participants. Upon completion of heteroplasmy estimates from previously extracted DNA, urine from an additional 4 individuals were sent to the original commercial lab for heteroplasmy testing.

Genomic and mitochondrial DNA were extracted from peripheral blood, and polymerase chain reactions (PCR) were conducted to amplify a segment of the *MT-ATP6* gene flanking m.9035 using a specific pair of oligodeoxynucleotide primers: MTATP6a-Fw 5’-CGGGGGTATACTACGGTCAA-3’ and MTATP6a-Rv 5’-GTTGTCGTGCAGGTAGAGG-3’. Amplified PCR products were sequenced at the UChicago Sanger Sequencing Core using BigDye V3.1 procedures and compared to an *MT-ATP6* complete genome reference sequence.

Heteroplasmy levels were obtained through the SnapGene software based on nucleotide levels at the m.9035 position. The percentages were calculated as the level of the cytosine mutation over all nucleotide counts at that position, for the forward and reverse sequencing reactions. Each patient sample was run twice with identical procedures. The reverse sequencing reaction had higher-quality reads, and the heteroplasmy was determined as the average of the two reverse sequences.

### Ethics

Approval for this study was obtained under either protocol 76-HG-0238 or 15-HG-1030 at NIH [24], protocol 14707A-CR017 at The University of Chicago Hospitals, and protocol 9702M12400 at the University of Minnesota Medical Center. Written informed consent was obtained from all participants.

## Results

### Literature Review

We identified seven prior reports containing individual level data on unique patients with the *MT-ATP6* c.9035 T > C variant. Findings are reported in tabular format in Table 2. 35 individuals in 10 families were described. All affected individualsh had homoplasmic or near homoplasmic levels of the variant. Ataxia was a ubiquitous clinical features. Developmental delay, spasticity, neuropathy, and ophthalmoplegia were also reported. Age of onset ranged from early childhood to adulthood. Brian MRI ranged from normal to cerebellar atrophy in reported cases, with elevated lactate on MRS reported in one individual. Biochemical labs including lactate, plasma amino acids, and GDF15 were reported to be abnormal in 2 individuals, normal in 4 individuals, and not reported in remaining cases. No retina or cardiac involvement were reported at individual level. We note these features were mentioned in large scale reviews of *MT-ATP6* patients, including the c.9035 T > C variant, reported in aggregate with other variants [6, 25].

**Table 2:** Review of ATP6 c.9035 T > C cases

Report	Cases	Families	Neurological findings	Other Findings	Age of onset	Imaging findings	Heteroplasmy (tissue tested)	Biochemical Lab Findings
Sikorska et al., 2009 [26]	16	1	Cerebellar ataxia, gait disturbance, developmental delay	NR	Childhood	Cerebellar atrophy, elevated lactate on MRS	Homoplasmic (blood)	NR
Pfeffer et al., 2012 [8]	3	1	Cerebellar ataxia, dysarthria, pyramidal signs	Short stature, speech delay	2–20	Cerebellar atrophy	90–96% (blood), 96% (muscle)	NR
Ng et al., 2019 [27]	8	3	Ataxia, peripheral neuropathy, pyramidal signs	Neuropathy	3–19	Cerebellar atrophy	> 90% (blood)	NR
Garret et al., 2019 [28]	2	1	Cerebellar ataxia	Learning disability, axonal neuropathy	20	NR	Near homoplastic (blood, urine)	NR
Haraux et al., 2019 [29]	1	1	Cerebellar ataxia	Psychomotor retardation, sensorimotor neuropathy	Childhood	NR	Homoplasmic (blood,muscle)	Mild elevation of lacate in blood and CSF
Capiau et al., 2022 [30]	2	2	Spastic ataxia, motor delay, dysarthria, hypertonia, epilepsy	Ophthalmoplegia	6mo-19	Cerebellar atrophy	Homoplasmic (Blood, fiborblast)	Normal urine organic acids, plasma amino acids, acylcarnitine profile
Lessard et al., 2025 [31]	3	1	Spastic paraplegia, cerebellar ataxia	Short stature	30–40	Cerebellar atrophy	83%-homoplasmic (blood)	Blood lactate, alaline, alanine:lysine ratio (4.1), and GDF15 elevated in one individual and normal in others

### Clinical Data

**Fig. 1:**
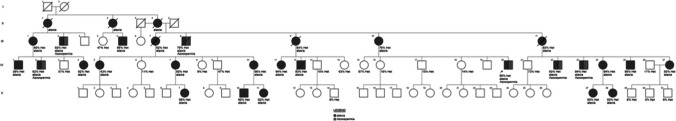
Pedigree and Genotype. Legend: Pedigree of a large extended family with ataxia and azoospermia due to the MT-ATP6 m.9035 T > C variant, along with heteroplasmy levels

A total of 45 individuals spanning three generations from a large extended family were included in this study (Fig. 1). Two individuals (IV-10 and IV-11) participated in the initial evaluations that led to the diagnosis. Patient IV-10 first noted gait changes at age 32 and was 67 at time of evaluation. IV-11 was noted ataxia symptoms at age 18 and was evaluated at 49. For both individuals, dilated eye exam showed no signs of retinitis or optic neuropathy. Echocardiogram was normal. Nerve conduction study showed mild axonal neuropathy for IV-11. Biochemical labs including lactate, pyruvate, ammonia, methylmalonic acid, carnitine and acylcarnitine profile, homocysteine level, plasma amino acids, and urine organic acids were unremarkable.

In all 30 individuals reported cerebellar ataxia affecting at least gait, while 15 did not report ataxia. Including the two cases mentioned above, full neurological examination records were obtained on 19 individuals while data on 26 others were obtained via phone interviews. The age of symptom onset among affected individuals ranged from 2 to 73 years, with a mean of 33 years and a median of 24 years. Clinical neurological evaluation in affected individuals revealed a combination of ataxia, oculomotor, pyramidal, and other findings (Table 3). Affected individuals all exhibited slowly progressive gait ataxia, with or without other signs, and had no clear episodic decompensations in the setting of metabolic stressors such as fevers or prolonged NPO states.

**Table 3:** Clinical characteristics

Pedigree	Sex	Heteroplasmy (blood)	Evaluation Age (unaffected)	Ataxia	Age of Ataxia	Oculomotor Signs	Pyramidal Tract Signs	Other Features	EMG/NCS
III:1	F	85.3%		Yes (G)	55	SI, OD	Hyporeflexia	S	
III:2	M	92.0%		Yes (G, L, D)	5	SI, N	Hyporeflexia, Spasicity, Babinski	SE, S, F,	Axonal Neuropathy
III:3	M		nr	No					
III:4	F	46.5%		No					
III:5	M	96.2%		Yes	2			SE	
III:7	F	61.5%		Yes (G, L, D)	63	SI, OD	Hyperreflexia		
III:8	M	78.6%		Yes (G, L, D)	30	SI, OD, N	Hyperreflexia	SE, S, F,	
III:9	F	83.7%		Yes (G)	56	SI	Hyperreflexia, Babinski	S	
III:10	F	76.4%		Yes (G)	70	SI	Hyperreflexia		
III:11	F	83.0%		Yes (G, L, D)	55	SI, N	Hyperreflexia, Babinski	SE, S, Masked Facies	
IV:1	M	39.1%		Yes	58				
IV:2	M	92.2%		Yes (G, L, D)	3	SI, OD, N	Hyporeflexia, Babinski	SE, S, F	Axonal Neuropathy
IV:3	M	51.0%		Yes	60				
IV:4	F	91.9%		Yes (G, L, D)	13	SI, OD, N	Hyporeflexia, Babinski	SE, S	Axonal Neuropathy
IV:5	F	43.2%		Yes	73				
IV:6	F	10.5%	71	No					
IV:7	F	85.0%		Yes	45				
IV:8	F	8.5%	nr	No					
IV:9	M	47.3%	66	No					
IV:10	F	96.0%		Yes (G, L, D)	12	SI, OD, N	Hyporeflexia, Babinski	S	Axonal Neuropathy
IV:11	F	93.9%		Yes (G, L)	18	SI, OD, N	Hyperreflexia	SE, S	Axonal Neuropathy
IV:12	M	93.4%		Yes (G, L, D)	7		Hyporeflexia	SE, S	Axonal Neuropathy
IV:13	M	10.3%	64	No					
IV:14	F	62.9%		Yes	61				
IV:15	F	86.8%	37	No					
IV:16	F	18.1%		No					
IV:17	M	15.1%	72	No					
IV:18	F	14.0%	76	No					
IV:20	M	89.8%		Yes (G, L, D)	24	SI, N	Hyperreflexia, Spasticity	S, F	Normal
IV:21	M	73.4%	61	No					
IV:22	M	92.5%		Yes (G, L, D)	13	SI, N	Hyporeflexia	SE, S, F	
IV:23	M	93.7%		Yes (G, L, D)	15	SI	Hyporeflexia, Babinski	SE, S, F	Axonal Neuropathy
IV:24	F	94.3%		Yes	55			SE	
IV:25	M	94.6%		Yes (G, L, D)	15	OD	Hyperreflexia	SE, S, F	
IV:26	M	10.7%		No					
IV:27	F	50.3%	nr	Yes	62			SE	
V:7	F	94.6%		Yes	16			SE	
V:10	M	94.6%		Yes (G, L, D)	13	SI, OD, N	Hyperreflexia, Babinski	SE	
V:11	F	92.1%		Yes (G, L, D)	12	SI, OD, N		SE	
V:14	M	8.8%	nr	No					
V:27	F	93.0%		Yes	22			SE	
V:28	F	92.9%		Yes	5			SE	
V:29	M	5.6%	45	No					
V:30	M	3.4%	44	No				SE	
V:31	M	5.6%	40	No				SE	

Legend: nr = not reported. Ataxia Column: G = Gait ataxia, L = limb ataxia, D = dysathria. Oculomotor Signs: SI = Saccadic intrusions during smooth pursuit, OD = ocular dysmetria, N = end gaze nystagmus. Pyramidal Tract Signs: Babinski = Extensor plantar reflexes. Other Features: SE = special education during school years, S = distal loss of vibration sense, F. = infertility

**Fig. 2:**
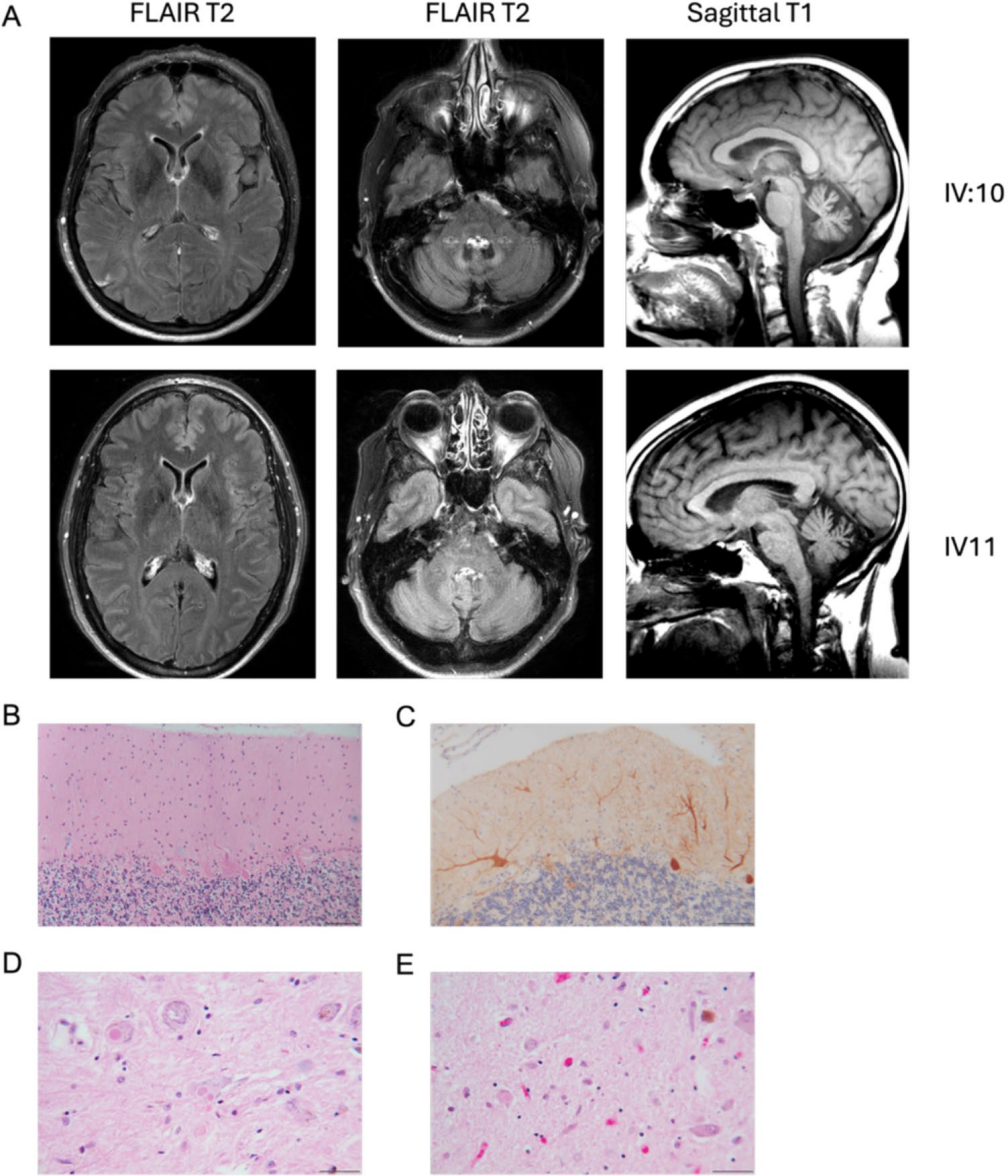
Brain Imaging and Pathology Findings. Legend: 2A: Representative MRI images including axial slices of the basal ganglia (left), axial slices of pons (middle), and sagittal midline in patient IV:10 and IV:11 demonstrated midline cerebellar atrophy. Images were taken on 3.0 T system. 2B: Superior cerebellar hemisphere with extensive loss of Purkinje neurons and “empty basket” fibers in the interface between the molecular layer and granular layer. One surviving Purkinje cell has a dilated proximal axon (torpedo). Hematoxylin and eosin. 2C: Calbindin immunohistochemical stain (brown) of superior cerebellar cortex showing abnormal dendritic morphology of surviving Purkinje cells. Calbindin immunohistochemistry (horseradish peroxidase-diaminobenzidine) with hematoxylin. 2D: Two neurons in substantia nigra with lewy bodies. 2E: Lewy bodies in neurons of locus coeruleus Scale bar = 100 µm

There we no reports of vision issues in anyone of this kindred, two of whom (IV:10 and IV11) had full retina evaluation. Similarly, no cardiac, muscle, hearing, or biochemical abnormalities could be attributed to this condition in our cohort. Brain MRI, done on 8 affected individuals, demonstrated midline predominant cerebellar atrophy (Fig. 2).

Brain autopsy was performed on III-11, revealing marked cerebellar cortical pathology, with variable loss of Purkinje neurons most prominent in the superior and anterior vermis and hemispheres. Numerous empty baskets, axonal torpedoes, and abnormal dendritic arborization of Purkinje cells were noted, while the dentate nucleus and cerebellar peduncles were preserved. Mild gliosis was seen in the inferior olives and subthalamic nuclei, and the spinal cord showed mild gracile tract degeneration. There was also severe loss of pigmented neurons and Lewy bodies in the substantia nigra pars compacta, with milder involvement of the locus coeruleus which was interpreated as comorbid Lewy Body Parkinson’s disease by pathology. (Fig. 2) Of note, this individual developed hypomimia later in life consistent with parkinsonism.

### Genotype

Genetic analysis was performed on 44 individuals, including all those reporting ataxia. Two index patients (IV-10 and IV-11) underwent clinical ataxia repeat expansion testing, research genome sequencing, and mitochondrial DNA sequencing, which revealed the pathogenic *MT-ATP6* c.9035 T > C variant at homoplasmic levels in blood. This variant was confirmed on clinical mtDNA testing. Segregation and heteroplasmy analysis were subsequently performed on 42 additional family members. Two additional individuals (IV-20 and V-11) had negative clinical repeat expansion testing previously independent of our study. Four additional individuals underwent confirmatory testing on fresh urine samples in a CLIA-certified laboratory, where heteroplasmy levels in both tissues correlated with the ranged derived from sanger sequencing data and relative to each other. Urine heteroplasmy levels were estimated to be between 50–75% in IV-14 (blood 62.9%), 52% in IV-5 (blood 43%), 5% in IV-6 (blood 10%), and 94% in IV-7 (blood 85%) with disclaimer that urine heteroplasmy estimates should within 25% of actual value by the commercial lab.

**Fig. 3:**
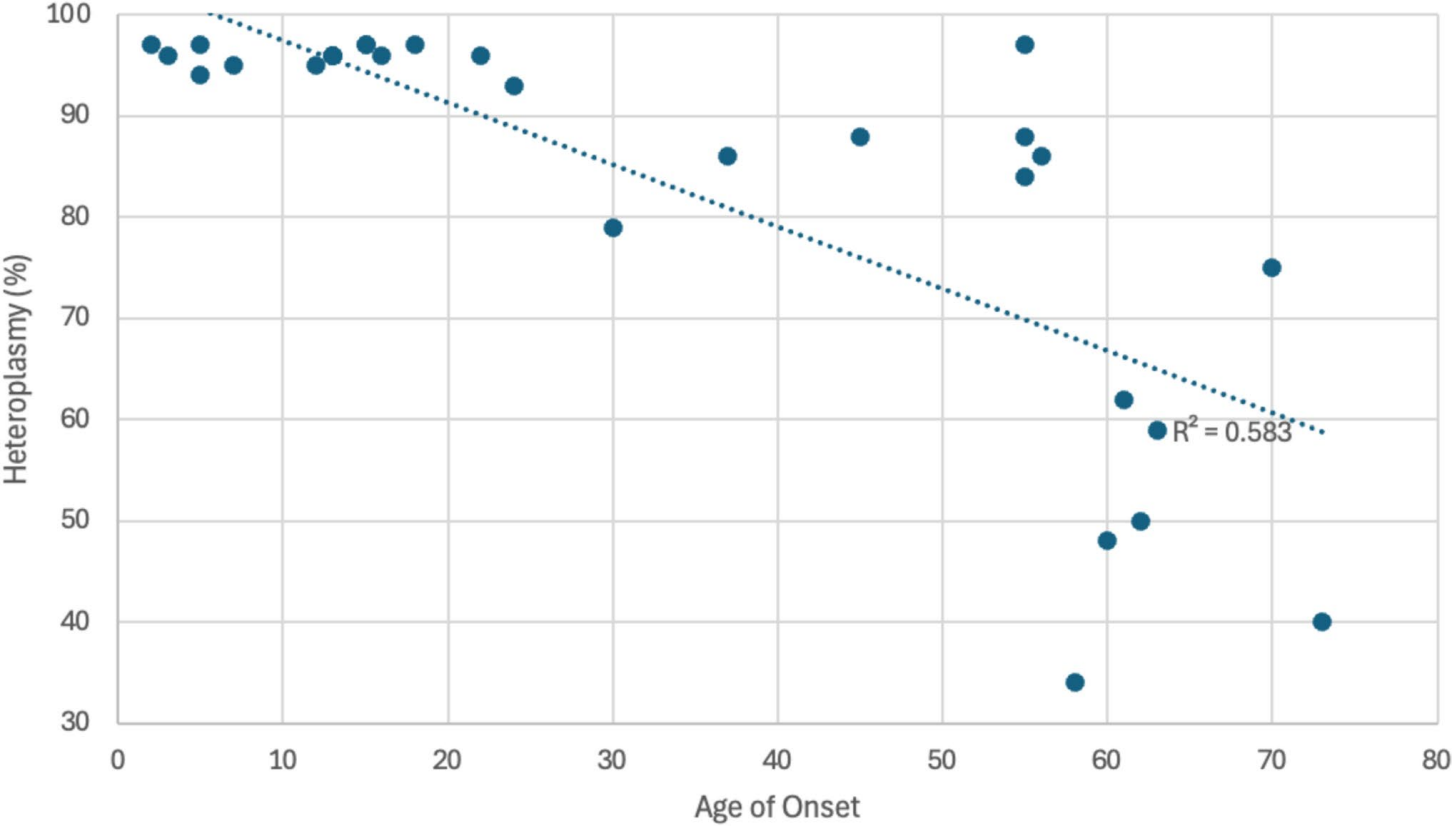
Heteroplasmy vs age of ataxia onset. Legend: Inverse correlation between heteroplasmy level and age of ataxia onset

Among unaffected individuals, heteroplasmy levels ranged from 3.4% to 73.4%, with an average of 19.8%. In contrast, affected individuals exhibited heteroplasmy levels ranging from 50.3% to 96.2%, with a mean of 92.5%. An inverse relationship was observed between heteroplasmy level and age of ataxia onset, although inter-individual variability was noted (Fig. 3). Specifically, all individuals with childhood-onset ataxia (onset < 18 years) had heteroplasmy levels ≥ 91%, with a mean of 93.4%. In contrast, those with adult-onset ataxia had a mean heteroplasmy level of 72.7%.

### Fertility

**Fig. 4:**
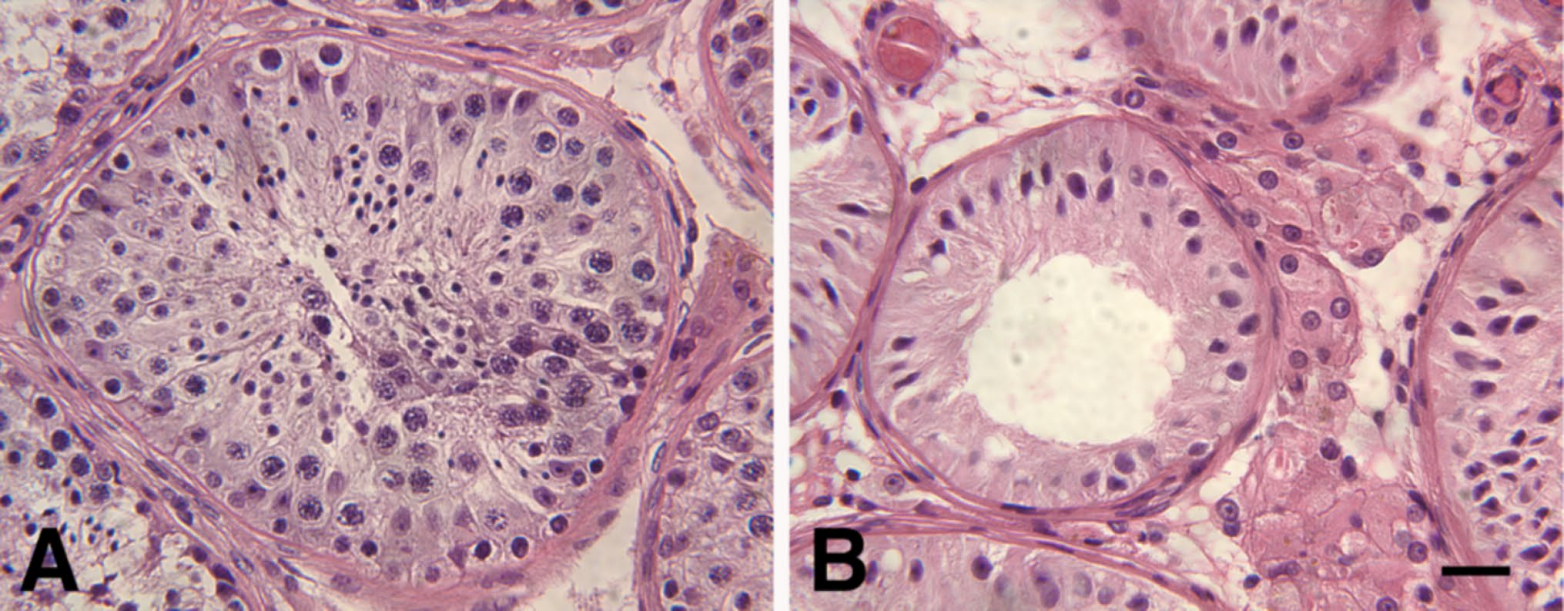
Absence of Testicular Germ Cells. Legend: A. Cross section of a normal seminiferous tubule showing normal sperm and precursors. B. Cross section of testicular biopsy from IV:22 with seminiferous tubules showing only Sertoli cells and lacking any sperm or precursors

None of the males affected by ataxia in this family had biological offspring. Among the seven affected males who underwent clinical fertility evaluation, all were diagnosed with infertility. Six were found to have testicular atrophy on exam. Semen analysis was completed on 6 of the 7, of which 5 were found ot have azoospermia and the sixth was asthenozoospermia. (Table 4). One individual (IV-22) had a testicular biopsy that showed absent germ cells (Fig. 4). A review of the literature revealed no prior reports of paternity among affected males with this mutation, suggesting a potential association with male infertility. However, the penetrance of this finding remains uncertain due to incomplete reproductive data across the cohort. No female member of this kindred reported fertility problems.

**Table 4:** Urological studies in males with ataxia and infertility

Pedigree #	Testicular Exam	Semen analysis	Testicular Biopsy
III:2	No testicular atrophy	Asthenozoospermia (50% sperm count w/95% nonmotile)	
III:8	Testicular atrophy	Azoospermia (No spermatozoa detected)	
IV:2	Testicular atrophy	Azoospermia (No spermatozoa detected)	
IV:20	Testicular atrophy	Azoospermia (No spermatozoa detected)	
IV:22	Testicular atrophy	Azoospermia (No spermatozoa detected)	No germ cells present
IV:23	Testicular atrophy	Azoospermia (No spermatozoa detected)	
IV:25	Testicular atrophy

### Cognitive

Seventeen of 30 individuals with ataxia (56.7%) and two of 15 unaffected individuals (13.3%) reported having received special education services during grade school (Table 3).

Within the thirteen affected family members who underwent neuropsychological testing (Ataxia Group), mean overall intellectual ability fell within the Low Average/Borderline Range (WAIS-R FSIQ), albeit with individual scores ranging from the Mild Intellectual Disability to Average Range. Similar performance levels were noted across subscales assessing verbal or visuospatial intellectual ability (WAIS-R VIQ and PIQ). Additional cognitive weaknesses noted within the Ataxia Group included limited ability to: (1) retain/mentally manipulate short strings of verbal information [auditory working memory], (2) utilize auditory working memory and sequential reasoning skills to process single or dual sets of familiar information [executive function], (3) encode and freely recall verbal information but intact recognition of the same information [memory retrieval deficit], (4) freely recall visual designs immediately following presentation, (5) rapidly produce words based on similar structural features [verbal fluency], and (6) execute motor tasks that required rapid repetitive finger movement or manual dexterity and speed to manipulate small objects was noted within the Ataxia Group (bilateral average performance score) (Table 5). Further evaluation of performance on the executive function task indicated significantly longer time to complete the more complex shifting pattern between sequential elements within two sets of information (Trailmaking Test B-Trailmaking Test A score; t (12) = −4.595, p < 0.001). However, the ratio of time to complete the dual set-shifting task/time to complete the single set shifting task was not statistically significant (Trailmaking Test B/Trailmaking Test A score; t (12) = −2.555, p = 0.025). Within the five unaffected family members (Unaffected Group), weaknesses in intellectual ability, verbal memory retrieval, reading comprehension, verbal fluency, and fine motor speed were noted but failed to reach a Family-Wise Error Rate (Bonferroni correction) level of statistical significance (Supplemental Table 1).

**Table 5:** Neuropsychological test performance in ataxia group

Ability Domain/Test	Z Score (Standard Deviation)	T Test (degrees freedom)	Probability Level
**Intellectual Ability**			
WAIS-R FSIQ	−1.34 (0.79)	−6.14(12)	< 0.001*
WAIS-R VIQ	−1.30 (0.83)	−5.66(12)	< 0.001*
WAIS-R PIQ	−1.31 (0.73)	−6.45(12)	< 0.001*
**Working Memory/Ideomotor Speed**			
WAIS-R Digit Span	−1.28 (0.76)	−6.11(12)	< 0.001*
Trailmaking Test A	−2.64 (2.75)	−3.47 (12)	0.002*
Trailmaking Test B	−3.91 (3.29)	−4.28(12)	< 0.001*
**Learning/Memory**			
AVLT Learning Across Trials	−1.98 (1.34)	−5.34(12)	< 0.001*
AVLT Immediate Recall	−1.43 (1.11)	−4.64(12)	< 0.001*
AVLT Delayed Recall	−1.25 (1.26)	−3.58(12)	0.002*
AVLT Delayed Recognition	0.13 (1.49)	0.30(12)	0.617
BVRT (# correct)	−2.05 (1.61)	−4.59(12)	< 0.001*
**Language Skills:**			
WJ-R Letter Word Identification	−0.86 (1.63)	−1.83(11)	0.047
WJ-R Passage Comprehension	−1.44 (1.62)	−2.95(10)	0.007
Verbal Fluency	−1.80 (1.18)	−5.50(12)	< 0.001*
**Fine Motor Skills:**			
Finger Tapping (Bilateral Average)	−3.44 (1.64)	−7.28(11)	< 0.001*
Grooved Pegboard (Bilateral Average)	−8.51 (8.08)	−3.65(11)	0.002*

*Significant with Bonferroni Correction

Legend: WAIS-R = Wechsler Adult Intelligence Scale-Revised, FSIQ = Full Scale Intellectual Quotient, VIQ = Verbal Intelligence Quotient, PIQ = Performance Intelligence Quotient, AVLT = Rey Auditory verbal Learning Test, BVRT = Benton visual Retention Test, WJ-R = Woodcock-John Tests of Achievement – Revised

Cognitive and motor function in the Ataxia Group was inversely correlated with heteroplasmy percent for several variables but reached a family wise error rate (Bonferroni correction) of statistical significance only for the relationship between heteroplasmy percent and intellectual ability (WAIS-R FSIQ, VIQ, and PIQ), ability to rapidly produce words based on similar structural features [Verbal Fluency], and rapid repetitive finger motion (Table 4). Age of ataxia onset was significantly correlated with measures of visuospatial intellectual ability (WAIS-R PIQ), verbal fluency, and finger tapping (Table 6).

**Table 6:** Correlation heteroplasmy level and cognitive/motor functions in ataxia group

	Heteroplasmy		Age of Onset	
	Pearson r (degrees freedom)	Probability Level*	Pearson r (degrees freedom)	Probability Level*
**Intellectual Ability:**				
WAIS-R FSIQ	−0.875 (11)	< 0.001*	0.768 (10)	0.004
WAIS-R VIQ	−0.823 (11)	< 0.001*	0.685 (10)	0.014
WAIS-R PIQ	−0.874 (11)	< 0.001*	0.835 (10)	< 0.001*
**Working Memory/Ideomotor Speed:**				
WAIS-R Digit Span	−0.620 (11)	0.024	0.705 (10)	0.011
Trailmaking Test A	−0.632 (11)	0.020	0.671 (10)	0.017
Trailmaking Test B	−0.545 (11)	0.054	0.691 (10)	0.013
**Learning/Memory:**				
AVLT Learning Across Trials	−0.626 (11)	0.022	0.716 (10)	0.009
AVLT Immediate Recall	−0.535 (11)	0.060	0.669 (10)	0.017
AVLT Delayed Recall	−0.448 (11)	0.125	0.659 (10)	0.020
AVLT Delayed Recognition	−0.233(11)	0.443	0.521 (10)	0.082
BVRT (#correct)	−0.489 (11)	0.090	0.717 (10)	0.009
**Language Skills:**				
WJ-R Letter Word Identification	−0.660 (10)	0.020	0.771 (9)	0.005
WJ-R Passage Comprehension	−0.699 (9)	0.017	0.746 (8)	0.013
Verbal Fluency	−0.792 (11)	0.001*	0.899 (10)	< 0.001*
**Fine Motor Skills:**				
Finger Tapping (Bilateral Average)	−0.863 (10)	< 0.001*	0.909 (9)	< 0.001*
Grooved Pegboard (Bilateral Average)	−0.564 (10)	0.056	0.711 (9)	0.014

*Statistically significant with Bonferroni correction

During brief clinical interviews before administration of neuropsychological tests, some individuals in both the Ataxia and Unaffected Groups reported a history of depressed mood, anxiety, anger management problems, or a history of psychoactive substance dependence. Two individuals reported a history of hallucinatory experiences. Within the Ataxia Group, emotional function indicators suggested a moderate, but statistically significant overall level of emotional dysfunction [GSI index], characterized by distress related to perception of and/or concern about somatic dysfunction, subjective cognitive weaknesses, mental/behavioral inflexibility, feelings of inadequacy/inferiority, depressed mood, subjective anxiety, specific fears, and social adjustment [self-referencing in social interactions, suspiciousness of others] (Table 7). Emotional function indicators were not significantly correlated with heteroplasmy level in the Ataxia Group (Supplemental Table 2). Statistically significant emotional concern/distress was not noted in the emotional function profile of the Unaffected Group (Supplemental Table 3).

**Table 7:** Emotional function indicators in ataxia group

**Emotional Function Domain**	Z Score (Standard Deviation)	T Score (degrees of freedom)	Probability Level*
**Global Severity Index (GSI)**	1.5 (0.8)	6.58 (12)	< 0.001*
Somatization Scale	1.3 (0.9)	5.43 (12)	< 0.001*
Obsessive–Compulsive Scale	1.6 (0.9)	6.67 (12)	< 0.001*
Interpersonal Sensitivity Scale	1.1 (1.0)	3.99 (12)	0.002*
Depression Scale	1.2 (1.1)	4.09 (12)	0.001*
Anxiety Scale	1.2 (1.2)	3.60 (12)	0.004*
Hostility Scale	0.9 (1.0)	3.19 (12)	0.008
Phobic Anxiety Scale	1.2 (0.9)	4.85 (12)	< 0.001*
Paranoid Ideation Scale	1.4 (1.2)	4.37 (12)	< 0.001*
Psychoticism Scale	1.1 (1.3)	2.97 (12)	0.012

*Statistically significant with Bonferroni correction

## Discussion

We report a large multigenerational family with a constellation of neurologic, cognitive, and reproductive phenotypes due to a pathogenic mtDNA variant in *MT-ATP6* (m.9035 T > C). Most individuals with ataxia in this pedigree harbored high heteroplasmy levels of the MT-ATP6 m.9035 T > C variant, with an inverse correlation between heteroplasmy and age of ataxia onset. Individuals with childhood-onset ataxia uniformly exhibited near-homoplasmic levels (> 91%), whereas those with adult-onset disease had lower heteroplasmy levels on average. While there is a clear correlation, no heteroplasmy threshold could be identified in this family. One subject (IV:21) had a relatively high heteroplasmy level (73.4%) but did not endorse ataxia at 60, which may reflect incomplete penetrance or a presymptomatic state. Conversely, two individuals (IV:1 and IV:5) had relatively low heteroplasmy levels (39% and 43% respectively) noted late ataxia onset at age 58 and 73. Possible explanations include tissue specific heteroplasmy differences since the brain was not tested or imprecise heteroplasmy measurements since these particular estimates were made based on sanger sequencing. It is also possible that these individuals have an alternative explanation for developing late onset ataxia though we were unable to identify a clear cause in their history and their genetic testing was limited to a repeat expansion panel.

A limitation of our study is in 26 cases we relied to patient reported status for clinical symptoms when an individual was unable to be examined in person or share previous medical records. Similarly we were not able to obtain sample to test every affected or potentially presymptomatic individual. We cannot rule out the possibility some individuals thought to be asymptomatic or presymptomatic may actually have signs of ataxia too mild to come to attention or some individuals reporting ataxia may have an alternative medical explanation. This may also have bias our ascertainment of individuals in later generations to only the most severely affected.

The primary phenotypes of slowly progressive cerebellar ataxia and borderline low or low cognition with variable pyramidal and sensory signs and absent retina or cardiac issues is consistent with prior reports of the m.9035 variant found on our review of the literature [8, 26]. Anticipation was observed in this kindred, with the mean age of ataxia onset decreasing from 42 in generation III to 35 in generation IV and 14 in generation V. This observation can likely be explained by a combination of highly variable heteroplasmy levels due to the mitochondrial bottleneck effect and ascertainment bias in disease recognition.

Brain MRI done on affected individuals showed cerebellar and pontine atrophy consistent with prior reports. Brain pathology reveals abnormal dendritic arborization of the cerebellar Purkinje cells consistent with previously reported pathology in cerebellar ataxia cases [32]. Purkinje cell degeneration is a frequent finding in most forms of hereditary ataxia and likely suggests a common vulnerability to a wide range of genetic/molecular/metabolic insults [33–36], including in the setting of primary mitochondrial disease [37]. Secondary mitochondrial dysfunction has also been associated with other forms of SCA such as SCA28 [38]. While parkinsonism has been reported in the setting of primarily mitochondrial diseases [39] and was previously reported in one case with an *mt-ATP6* variant [40], this finding was not described in previously published cases with the m.9035 T > C variant. We agree with the clinical interpretation that depletion of pigmented neurons and presence of Lewy bodies in the substantia nigra seen in our patient is related comorbid Lewy-body Parkinson’s disease and do not claim this finding suggests Parkinson’s pathology is part of the *mt-ATP6* related phenotype.

In addition to cerebellar ataxia, many affected individuals reported early learning difficulties and participation in special education programs. This is consistent with prior reports in our review of the literature. In the present study, cognitive testing, administered to a subset of family members who had been diagnosed with ataxia, suggested a number of cognitive weaknesses, including intellectual ability ranging from the Mild Intellectual Disability to Average Range, as well as limited auditory working memory, visual sequential reasoning/executive function, verbal memory retrieval, visual memory, verbal fluency, and fine motor skills. Although the performance profile of unaffected family members suggested possible weaknesses in verbal intellectual ability, verbal memory retrieval, reading comprehension, and repetitive fine motor speed, findings failed to reach a level of statistical significance.

During a brief clinically non-diagnostic interview prior to administration of cognitive tests, some individuals reported a history of depressed mood, anxiety, anger management problems, hallucinatory experiences, and/or psychoactive substance dependence. Among family members diagnosed with ataxia, significant emotional dysfunction characterized by subjective perception of somatic and cognitive weakness, feelings of inadequacy/inferiority, social adjustment problems, and mood dysregulation was suggested by significant scale elevations on a brief self-report measure of psychiatric dysfunction. In contrast, significant psychiatric problems were not noted in the psychiatric self-report profiles of unaffected family members.

Heteroplasmy level within the ataxia group was inversely correlated with overall, verbal, and visuospatial intellectual ability, verbal fluency, and repetitive motor speed. A subset of these functional deficits, including visuospatial intellectual ability, verbal fluency, and repetitive motor speed were also found to be significantly correlated with age of ataxia onset. Scores on a self-report measure of psychiatric dysfunction were elevated at a statistically significant level only within the ataxia group and were uncorrelated with heteroplasmy level.

An associative relationship between indices of disease severity and selected performance variables, noted only within the Ataxia Group, suggests a broader cognitive-motor component to this mitochondrial disease ataxia phenotype. In contrast, results of this study provide no strong evidence of a similar relationship between psychiatric dysfunction and disease phenotype. Psychiatric adjustment issues were reported nonselectively during a non-diagnostic clinical interview by affected and unaffected family members. Significant scale elevations were noted within the ataxia group but not among unaffected family members on a brief self-assessment measure of psychiatric problems. However, ataxia group heteroplasmy levels were uncorrelated with self-reported emotional dysfunction on the psychiatric self-report inventory. Given small sample sizes in this study of the relationship between cognitive/psychiatric manifestations and disease phenotype within the present study, validation of these findings with larger samples in future studies will be essential.

Deficits in auditory working memory, executive function, visuospatial function, verbal fluency, auditory memory retrieval, and emotional dysfunction have been noted in some studies of spinocerebellar ataxia and cerebellar lesions and cited as evidence of a Cerebellar Cognitive Affective Syndrome related to dysfunction in cerebellar-cortical neural circuits [41]. However, neuropsychological tests of complex cognitive function (executive function) are multi-dimensional and sensitive to performance adequacy in several other cognitive/motor domains. For example, while deficient performance on a test assumed to reflect executive dysfunction (Trail Making Test) was noted in this study, further evaluation of the profile suggested that demands placed on visuospatial search and visuomotor integration speed skills contributed significantly to weak performance on cognitively simpler and more complex components of this task within the Ataxia Group. Prior studies of spinocerebellar ataxia have demonstrated weak performance on measures of repetitive motor speed and dexterity and kinematic studies of repetitive finger tapping have documented slower speed and increased variability of movement, especially when higher rates of responding are required [42–46]. It is unclear whether the cognitive/motor dysfunction noted within the ataxia group in the present study are part of the primary mitochondrial disease pathology or related to cerebellar dysfunction. [41]

A particularly notable observation is the apparent association between the *MT-ATP6* m.9035 T > C variant and male infertility. None of the males with clinical ataxia in this family had biological offspring, and formal fertility evaluations in seven individuals revealed azoospermia or severely impaired sperm motility. While infertility has not previously been described in *MT-ATP6*-related disease in humans, mitochondrial function and variants in *MT-ATP6* in particular have been linked to sperm health in both human and non-human studies [47–49]. Moreover, on review of previously published families with ataxia due to the m.9035 T > C variant, no affected males were reported to have offspring, suggesting male infertility may be an underrecognized feature [8, 26]. The penetrance of this finding remains unclear since comprehensive reproductive histories were not available for all at-risk males.

Also of note, affected individuals in this family did not exhibit many of the multi-systemic features previously reported in patients with *MT-ATP6* related disease [6, 7, 25, 50] including retinopathy, myopathy, cardiac disease, and biochemical abnormalities. In the two largest previous reports on *MT-ATP6* patients containing 218 cases [6] and 111 cases [25], only 13 and 1 individual respectively harbored the m.9035 T > C variant. Affected patients with the m.9035 T > C variant may be under-represented in MT-ATP6 studies based in pediatric centers. Our findings, including our review of the literature and studies done on this family, suggest the m9035T > C variant predominately cause a neurodegenerative phenotype without cardiac or retina findings and with inconsistent biochemical findings.

## Conclusion

Taken together, this study suggests male infertility has a potentially under-recognized phenotype of *MT-ATP6*-related disease. It remains to be determined whether male infertility is specific to the c.9035 T > C variant or might be observed more broadly across other pathogenic *MT-ATP6* mutations. Further investigation of fertility status and sperm function in additional families and cohorts with mtDNA variants is warranted to clarify the scope and mechanism of reproductive involvement in mitochondrial disease.

While cerebellar ataxia and neurodegeneration are frequent phenotypes in primary mitochondrial disease [51], there is limited data on the yield of mtDNA sequencing for families with cerebellar ataxia. One recent study on the yield of genome sequencing for hereditary cerebellar ataxia found a causative mtDNA mutation in 2% of cases [52]. This family also underscores the importance of getting mtDNA testing for patients with adult-onset ataxia, even the presence of typical features of spinocerebellar ataxias such as apparent anticipation.

## Supplementary Information

Below is the link to the electronic supplementary material.

Supplementary file1 (DOCX 17 KB)

Supplementary file2 (DOCX 15 KB)

Supplementary file3 (DOCX 15 KB)

## Data Availability

All data supporting the findings of this study are available within the paper and its Supplementary Information.
