# Supplementary material for: MT-ATP6 9035T>C Variant Causes Ataxia With Azoospermia and Apparent Anticipation in a Four-generation Kindred
Source: Cerebellum. 2026 Apr 25;25(3):61. doi: 10.1007/s12311-026-02008-z (PMC13110232; doi:10.1007/s12311-026-02008-z)
Supplement: Supplementary file 1 — Supplementary file1 (DOCX 17 KB) [file 12311_2026_2008_MOESM1_ESM.docx]

Supplemental 1: Neuropsychological Test Performance in Unaffected Group

| **Ability Domain/Test** | **Z Score/Standard Deviation** | **T Test (degrees freedom)** | **Probability Level*** |
| --- | --- | --- | --- |
| **Intellectual Ability** |  |  |  |
| WAIS-R FSIQ | -0.92 (1.12) | -1.84(4) | 0.070 |
| WAIS-R VIQ | -0.99 (0.87) | -2.53(4) | 0.032 |
| WAIS-R PIQ | -0.89 (1.19) | -1.68(4) | 0.084 |
|  |  |  |  |
| **Working Memory/Ideomotor Speed** |  |  |  |
| WAIS-R Digit Span | -0.40 (1.32) | -0.68(4) | 0.268 |
| Trailmaking Test A | -0.88 (2.85) | -0.69(4) | 0.263 |
| Trailmaking Test B | -1.16 (2.27) | -1.14(4) | 0.159 |
|  |  |  |  |
| **Learning/Memory** |  |  |  |
| AVLT Learning Across Trials | -1.56 (1.26) | -2.77(4) | 0.025 |
| AVLT Immediate Recall | -0.98 (0.46) | -4.81(4) | 0.004 |
| AVLT Delayed Recall | -1.13 (0.85) | -2.97(4) | 0.021 |
| AVLT Delayed Recognition | 0.52 (0.39) | 3.01(4) | 0.980 |
|  |  |  |  |
| BVRT (# correct) | -0.24 (0.94) | -0.499(4) | 0.326 |
|  |  |  |  |
| **Language Skills:** |  |  |  |
| WJ-R Letter Word  Identification | -0.68 (1.84) | 0.013(4) | 0.228 |
| WJ-R Passage  Comprehension | -1.17 (1.21) | -2.17(4) | 0.048 |
| Verbal Fluency | -0.81 (0.85) | -2.12(4) | 0.051 |
| **Fine Motor Skills:** |  |  |  |
| Finger Tapping (Bilateral  Average) | -2.52 (1.63) | -2.95(4) | 0.021 |
| Grooved Pegboard (Bilateral  Average) | -1.83 (2.68) | -1.52(4) | 0.101 |

*None significant with Bonferroni correction
