# Supplementary material for: MT-ATP6 9035T>C Variant Causes Ataxia With Azoospermia and Apparent Anticipation in a Four-generation Kindred
Source: Cerebellum. 2026 Apr 25;25(3):61. doi: 10.1007/s12311-026-02008-z (PMC13110232; doi:10.1007/s12311-026-02008-z)
Supplement: Supplementary file 2 — Supplementary file2 (DOCX 15 KB) [file 12311_2026_2008_MOESM2_ESM.docx]

Supplemental Table 2: Correlation Heteroplasmy Level with Emotional Function Variables in Ataxia Group

| **Emotional Function Domain** | Pearson r (degrees freedom) | Probability Level* |
| --- | --- | --- |
| **Global Severity Index (GSI)** | 0.445 (11) | 0.127 |
| Somatization Scale | 0.558 (11) | 0.047 |
| Obsessive-Compulsive Scale | 0.068 (11) | 0.825 |
| Interpersonal Sensitivity Scale | 0.189 (11) | 0.535 |
| Depression Scale | 0.471 (11) | 0.104 |
| Anxiety Scale | 0.325 (11) | 0.279 |
| Hostility Scale | 0.306 (11) | 0.310 |
| Phobic Anxiety Scale | 0.487 (11) | 0.092 |
| Paranoid Ideation Scale | 0.339 (11) | 0.257 |
| Psychoticism Scale | 0.306 (11) | 0.310 |

*None significant with Bonferroni correction
