# Supplementary material for: MT-ATP6 9035T>C Variant Causes Ataxia With Azoospermia and Apparent Anticipation in a Four-generation Kindred
Source: Cerebellum. 2026 Apr 25;25(3):61. doi: 10.1007/s12311-026-02008-z (PMC13110232; doi:10.1007/s12311-026-02008-z)
Supplement: Supplementary file 3 — Supplementary file3 (DOCX 15 KB) [file 12311_2026_2008_MOESM3_ESM.docx]

Supplemental 3: Emotional Function Indicators in Unaffected Group

| **Emotional Function Domain** | Z Score (Standard Deviation) | T Score (degrees of freedom) | Probability Level |
| --- | --- | --- | --- |
| **Global Severity Index (GSI)** | 1.1 (1.4) | 1.5 (3) | 0.115 |
| Somatization Scale | 0.7 (1.5) | 0.88 (3) | 0.221 |
| Obsessive-Compulsive Scale | 1.6 (1.0) | 3.07 (3) | 0.027 |
| Interpersonal Sensitivity Scale | 1.1 (1.5) | 1.42 (3) | 0.125 |
| Depression Scale | 0.8 (1.7) | 0.93 (3) | 0.211 |
| Anxiety Scale | 0.7 (1.5) | 0.95 (3) | 0.206 |
| Hostility Scale | 1.0 (1.5) | 1.35 (3) | 0.135 |
| Phobic Anxiety Scale | 0.7 (1.2) | 1.11 (3) | 0.174 |
| Paranoid Ideation Scale | 0.4 (1.4) | 0.59 (3) | 0.298 |
| Psychoticism Scale | 1.1 (1.4) | 1.51 (3) | 0.114 |
